# Analysis of Immune Cell Subsets in Peripheral Blood from Patients with Engineered Stone Silica-Induced Lung Inflammation

**DOI:** 10.3390/ijms25115722

**Published:** 2024-05-24

**Authors:** Gema Jiménez-Gómez, Antonio Campos-Caro, Alejandro García-Núñez, Alberto Gallardo-García, Antonio Molina-Hidalgo, Antonio León-Jiménez

**Affiliations:** 1 Biomedical Research and Innovation Institute of Cadiz (INiBICA), 11009 Cadiz, Spain; jggema@gmail.com (G.J.-G.); hpayns@gmail.com (A.G.-N.); antoniohmolina@hotmail.com (A.M.-H.); antonio.leon.sspa@juntadeandalucia.es (A.L.-J.); 2 Research Unit, Puerta del Mar University Hospital, 11009 Cadiz, Spain; 3 Genetics Area, Biomedicine, Biotechnology and Public Health Department, School of Marine and Environmental Sciences, University of Cadiz, 11510 Cadiz, Spain; 4 Immunology Department, Puerta del Mar University Hospital, 11009 Cadiz, Spain; bertogallar@yahoo.es; 5 Pulmonology Department, Puerta del Mar University Hospital, 11009 Cadiz, Spain

**Keywords:** immune cell subsets, lymphocytes, artificial stone, silicosis, human

## Abstract

Silicosis caused by engineered stone (ES-silicosis) is an emerging worldwide issue characterized by inflammation and fibrosis in the lungs. To our knowledge, only a few reports have investigated leukocyte/lymphocyte subsets in ES-silicosis patients. The present study was designed to explore the proportions of the main lymphocyte subsets in ES-silicosis patients stratified into two groups, one with simple silicosis (SS) and the other with a more advanced state of the disease, defined as progressive massive fibrosis (PMF). The proportions of B (memory and plasmablasts) cells, T (helper, cytotoxic, regulatory) cells, and natural killer (NK) (regulatory and cytotoxic) cells were investigated by multiparameter flow cytometry in 91 ES-silicosis patients (53 SS patients and 38 PMF patients) and 22 healthy controls (HC). Although the total number of leukocytes did not differ between the groups studied, lymphopenia was observed in patients compared to healthy controls. Compared with those in healthy controls, the proportions of memory B cells, naïve helper T cells, and the CD4^+^/CD8^+^ T cells’ ratio in the peripheral blood of patients with silicosis were significantly decreased, while the percentages of plasma cells, memory helper T cells, and regulatory T cells were significantly increased. For the NK cell subsets, no significant differences were found between the groups studied. These results revealed altered cellular immune processes in the peripheral blood of patients with ES-silicosis and provided further insight into silicosis pathogenesis.

## 1. Introduction

Silicosis is a debilitating and incurable lung disease diagnosed in workers associated with exposure to crystalline silica (CS) particles from multiple sources, such as mining, construction (rock drilling, cutting, quarrying), sandblasting, and pottery polishing [1,2]. Recently, workers who have been working (cutting, sawing, polishing) with artificial silica agglomerates or engineered stones (ESs) used mainly in the construction of countertops in baths and kitchens have been diagnosed with silicosis disease worldwide. Silicosis due to CS exposure from engineered stone (ES-silicosis) is characterized by a short latency period and extensive pulmonary damage, and the disease typically occurs in young workers [3,4]. In addition, the progression from simple chronic silicosis (SS) to progressive massive fibrosis (PMF) of this entity continues even after cessation of exposure to silica [5].

It is thought that the pathophysiology of ES-silicosis starts with the deposition of CS particles into alveoli, where they are ingested by alveolar macrophages and, due to their inability to eliminate them, an inflammatory reaction occurs in which the lung epithelial cells and the immune system are involved [6,7,8].

The role of the immune system in the initial steps and in the progression of the disease is not completely understood. Leukocytes are known to modulate inflammation and fibrosis through the secretion of cytokines, but very little data have focused on lymphocyte subset characterization in peripheral blood from silicosis patients [9,10,11,12,13,14] and no such data have been obtained for ES-induced silicosis patients. Previously, we analysed the leukocyte subpopulations in ES-silicosis but not the lymphocyte subsets [15]. Furthermore, some discordant data have been reported regarding the variation in the total number of lymphocytes in silicosis patients and in nonsilicosis workers exposed to CS [12,14,15,16].

We hypothesized that a more detailed characterization of the main blood lymphocyte subsets (B lymphocytes, T lymphocytes, and NK lymphocytes) can provide new insights into the pathogenesis of ES-silicosis. Accordingly, the objective of the present study was to examine the levels of the main lymphocyte subsets in the peripheral blood of ES-silicosis patients diagnosed with different stages of the disease, SS and PMF. In parallel, a healthy control group not exposed to CS was analysed. Our findings revealed progressive lymphopenia in the SS and PMF groups compared to the healthy group, with lower counts of memory B lymphocytes and a lower CD4^+^/CD8^+^ T cell ratio but a greater percentage of regulatory T cells (Tregs) and plasmablasts.

## 2. Results

### 2.1. Characteristics of the Study Population

A total of 91 patients with silicosis agreed to participate in the study, of whom 53 were diagnosed with SS and 38 with PMF. All the subjects studied were males, Caucasians, and originated from the same geographic area (province of Cadiz, Spain); their sociodemographic data are shown in Table 1. The mean age, starting age, and duration of exposure to engineered stone dust were similar, without significant differences between the groups studied. A healthy control (HC) group of 22 volunteers with sociodemographic characteristics similar to those of patients, but not exposed to silica dust, was also studied.

### 2.2. Analysis of Cell Populations in Peripheral Blood from Patients and Healthy Controls

#### 2.2.1. Leukocyte Populations

**Figure 1:**
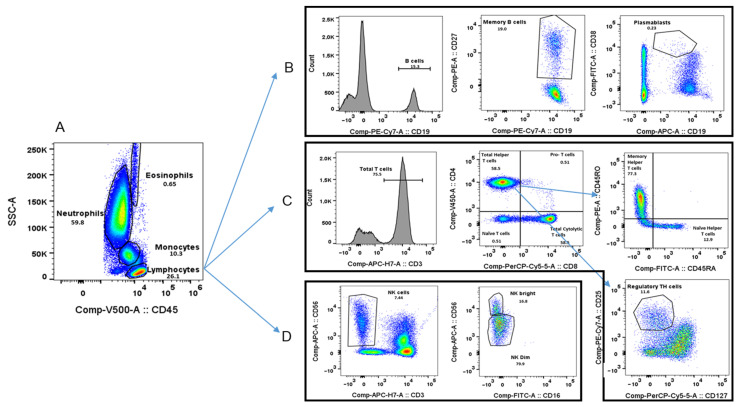
Flow cytometry detection of main B lymphocytes, T lymphocytes, and NK cell subsets. A representative plot (using a side-scatter (SSC) versus CD45 plot) for leukocyte populations from a healthy control volunteer is shown (**A**). Lymphocyte gate representative histograms or dot plots for B cell (**B**), T cell (**C**), and NK cell (**D**) subsets are shown.

**Figure 2:**
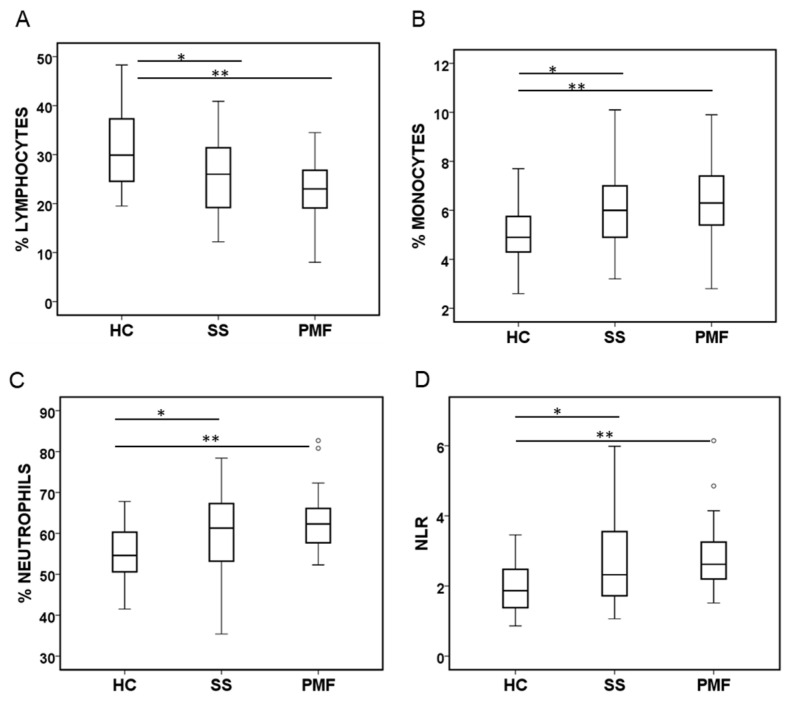
Main leukocyte cell populations quantified by flow cytometry in peripheral blood from patients diagnosed with simple silicosis (SS) or pulmonary massive fibrosis (PMF) and healthy controls (HC). (**A**) Percentage of lymphocytes. (**B**) Percentage of monocytes. (**C**) Percentage of neutrophils. (**D**) Neutrophil/lymphocyte ratio (NLR). ° indicate outliers. * and ** indicate significance at *p* < 0.05 and *p* < 0.01, respectively.

To determine whether the cell populations present in the peripheral blood of silicosis patients could reflect their disease severity, we analysed the main leukocyte subtypes by flow cytometry based on the labelling of cells with CD45 and the side-scatter parameter [17]. A representative dot plot of the flow cytometry analysis is shown in Figure 1. As expected, the numbers of major cell subtypes previously obtained with a haemocytometer counter from routine clinical blood tests [15] correlated well with the cell numbers obtained with flow cytometry (Supplementary Figure S1). The total white blood cell count did not differ among the HC, SS, or PMF groups. However, some differences were observed between the groups when lymphocyte, neutrophil, or monocyte numbers were compared (Figure 2A–C). Thus, when either of the patient groups, SS or PMF, were compared with the HC group, a statistically significant decrease in the percentage of lymphocytes and, on the contrary, an increase in the monocyte and neutrophil percentages were observed. Recently, the neutrophil/lymphocyte ratio (NLR) has been described as a biomarker for lung interstitial disease [18,19], and as expected, in line with our lymphocyte and neutrophil count results, the NLR was also significantly elevated in any of the SS and PMF silicosis groups compared to HC group (Figure 2D).

#### 2.2.2. Exploring the B Cells

**Figure 3:**
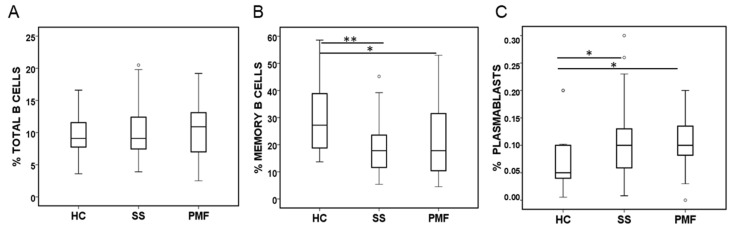
B cell subsets quantified by flow cytometry in peripheral blood from patients diagnosed with simple silicosis (SS) or pulmonary massive fibrosis (PMF) and healthy controls (HC). (**A**) Percentage of total B cells (CD19^+^). (**B**) Percentage of memory B cells (CD19^+^CD27^+^). (**C**) Percentage of plasmablasts (CD38^++^CD19^+/−^). ° indicate outliers. * and ** indicate significance at *p* < 0.05 and *p* < 0.01, respectively.

A further analysis of lymphocyte subtypes revealed some differences between the study groups. First, regarding the B cell lineage, the total B cell percentage (CD19^+^) was similar among the HC, SS, and PMF groups (Figure 3A), but the percentage of the memory B cell subset (CD19^+^CD27^+^) was lower in the SS and PMF groups than in the HC group (Figure 3B). In contrast, the number of plasma cells (CD38^++^CD19^+/−^) was greater in both silicosis groups than in the HC group (Figure 3C). Statistically, no significant differences were observed between the SS and PMF groups.

#### 2.2.3. Exploring the T Cells

**Figure 4:**
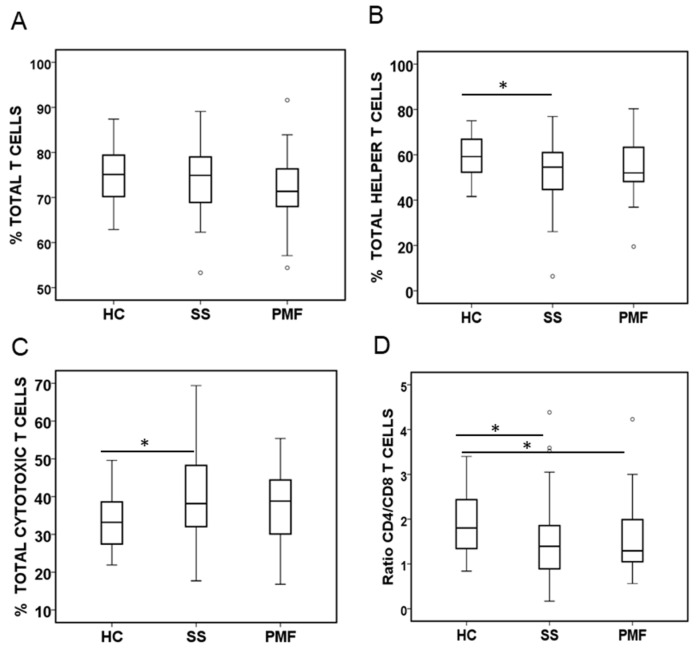
Total T cells and CD4^+^, CD8^+^ subsets quantified by flow cytometry in peripheral blood from patients diagnosed with simple silicosis (SS) or pulmonary massive fibrosis (PMF) and healthy controls (HC). (**A**) Percentage of total T cells (CD3^+^). (**B**) Percentage of T helper cells (CD3^+^CD4^+^). (**C**) Percentage of cytotoxic T cells (CD3^+^CD8^+^). (**D**) CD4^+^/CD8^+^ ratio. ° indicate outliers. * indicate significance at *p* < 0.05.

An analysis of the percentage of the main T lymphocyte subsets revealed that the mean percentage of total T lymphocytes (CD3^+^) tended to decrease, although not significantly so, between the HC and silicosis groups (Figure 4A). However, the percentage of T helper lymphocytes (CD3^+^CD4^+^) was significantly lower in the SS patient group than in the HC group (Figure 4B), and, in contrast, the percentage of cytotoxic T lymphocytes (CD3^+^CD8^+^) was significantly greater in the SS patient group than in the HC group (Figure 4C). Although the trend was the same for the PMF group, no significant differences were observed between this group and the HC or the SS group. Therefore, to emphasize the relationship between these two subpopulations, the CD4^+^/CD8^+^ T cells’ ratio was calculated, and this ratio was significantly lower in both silicosis patient groups than in the HC group (Figure 4D).

**Figure 5:**
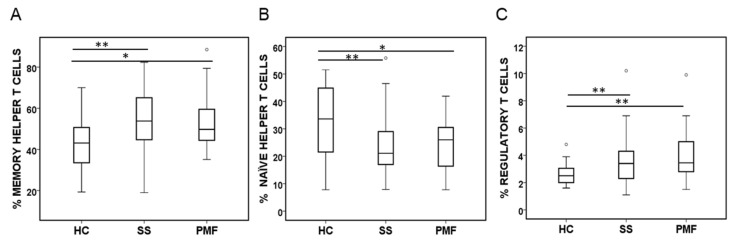
Some CD4^+^ T cell subsets quantified by flow cytometry in peripheral blood from patients diagnosed with simple silicosis (SS) or pulmonary massive fibrosis (PMF) and healthy controls (HC). (**A**) Naïve T helper cells (CD3^+^CD4^+^CD45RA^+^CD45RO^−^). (**B**) Memory T helper cells (CD3^+^CD4^+^CD45RA^−^CD45RO^+^). (**C**) Percentage of regulatory T cells (CD3^+^CD4^+^CD25^high^CD127^low/−^). ° indicate outliers. * and ** indicate significance at *p* < 0.05 and *p* < 0.01, respectively.

Other Th cell subsets were further analysed. The percentage of naïve Th cells (CD3^+^CD4^+^CD45RA^+^CD45RO^−^) was significantly lower in the samples from patients with silicosis in both the SS and PMF groups than in those from the HC group (Figure 5A). However, the percentage of memory Th cells (CD3^+^CD4^+^CD45RA^−^CD45RO^+^) was significantly increased in the silicosis groups compared to the HC group (Figure 5B). Tregs are considered to play an essential role in regulating the immune cellular response during an insult/inflammation, and, for this reason, CD3^+^CD4^+^CD25^high^CD127^low/−^ cells [20,21] were also analysed. Among the groups studied, a significant gradual increase in the percentage of these cells’ order of PMF > SS > HC was observed, although the increase was significant between the HC group and both of the silicosis groups (Figure 5C).

#### 2.2.4. Exploring the NK Cells

**Figure 6:**
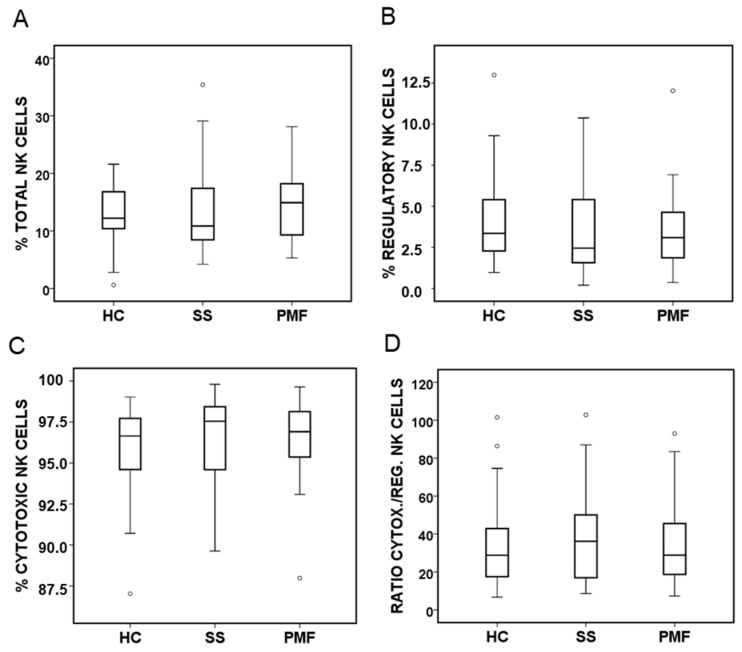
NK cell subsets quantified by flow cytometry in peripheral blood from patients diagnosed with simple silicosis (SS) or pulmonary massive fibrosis (PMF) and healthy controls (HC). (**A**) Percentage of total NK cells (CD56^+^). (**B**) Percentage of regulatory NK cells (CD56^bright^CD16^low/−^). (**C**) Percentage of cytotoxic NK cells (CD56^dim^CD16^+^). (**D**) Regulatory/cytotoxic NK cell ratio. ° indicate outliers.

The percentage of total NK cells in the lymphocyte fraction was calculated. However, no differences were found between the groups analysed (Figure 6A). With respect to NK cell subsets, we studied the two main populations, the main effector population (CD56^dim^CD16^+^) and the main regulatory population (CD56^bright^CD16^low/−^), and there were no differences between any of the groups (Figure 6B,C). We also did not observe differences in the cytotoxic/regulatory ratio between the studied groups (Figure 6D).

## 3. Discussion

This work was undertaken to explore the possible alterations in peripheral blood immune cell subsets in a well-known and broad group of patients with silicosis caused by engineered stone [5]. Although the ideal would have been to study lung samples through bronchoalveolar lavage, an analysis of blood cells allows us to obtain valuable information with minimal risk to the patients. The results of this study demonstrate and corroborate a progressive decrease in the number of lymphocytes and a progressive increase in the number of monocytes and neutrophils when comparing an HC group with a group diagnosed with SS and a group diagnosed with PMF, respectively, by using two different instruments, a research instrument (flow cytometer) and a clinical instrument (haematology analyser) [15].

One of the findings that emerged from this study was progressive lymphopenia in patients with ES-silicosis. There are some controversial results in the literature in this regard. On one hand, according to our results, several studies have reported lymphopenia in CS-exposed silicosis patients [10,12,14], but other works have not reported lymphopenia in CS-exposed nonsilicosis patients [16] or in silicosis patients [22]. Some of these discrepancies may be due to the nature of the origin of the crystalline silica to which the participants in the different studies were exposed, to the sex and/or age of the participants, or to the duration of the exposure.

More specifically, in the lymphocyte subsets studied in this work, we did not observe changes in the percentage of total B lymphocytes, similar to the findings of a previous study [12], which reported that the number of B cells, but not their percentage in the lymphocyte population, was reduced in silicosis patients compared to controls. We observed a clear decrease in the percentage of memory B cells; to our knowledge, this has not been reported before in silicosis patients but it has been described in systemic sclerosis where memory B cells have a high expression of CD95, which could explain an increase in apoptosis and therefore could explain a decrease in this cell subset [23]. A reduction in the number of memory B cells could contribute to an increase in the risk of autoimmune disease [24,25], as it is assumed to occur in silicosis patients [26] and patients with other diseases. On the other hand, despite the reduced number of memory B cells in patients with ES-silicosis and the usually low number of circulating plasmablasts in the peripheral blood of healthy controls, we observed a significant increase in plasmablasts in patients with ES-silicosis. Considering plasmablasts as immunoglobulin producers, this may be in line with the increase observed in the levels of immunoglobulins reported in other works [10,27,28], although the opposite has also been described [13]. An important role of certain B lymphocyte subsets in the development of silica-induced lung inflammation and fibrosis has been reported in humans and mice [29,30,31]. A more expanded analysis of all B cell subsets could provide more information about the role of these cells in patients with ES-silicosis.

In the present study, within the T cell compartment, no significant differences were observed in total CD3^+^ T cells, but there was a decrease in the percentage of CD4^+^ T cells and an increase in the percentage of CD8^+^ T cells; these differences are better reflected in the CD4^+^/CD8^+^ ratio. Our results are consistent with findings observed in several studies with patients with silicosis [32,33] and partially fit with the results presented by others in which only a reduction in CD4^+^ T cells but no changes in CD8^+^ T cells or in the CD4^+^/CD8^+^ T cell ratio were observed [10,13]. Additionally, other studies have documented other differences in these populations, such as a decrease in CD3^+^, CD4^+^, and CD8^+^ but no difference in the CD4^+^/CD8^+^ ratio [34] or a decrease that covers the number of cells of all the populations studied, but not their percentages relative to the total lymphocytes [12].

Naïve Th cells (CD4^+^CD45RA^+^) are considered an important part of the immune system. Variation in the size of the naïve T cell population can determine the magnitude of the T cell response [35]. We observed a significant reduction in patients with ES silicosis compared to the HC group, but, unfortunately, there are practically no studies that describe this cell population in patients with silicosis except for one study in which a decrease in their number was observed in patients with silicosis [9]. The decrease in the number of naïve Th cells correlated well with the increase in the number of memory Th cells (CD4^+^CD45RO^+^) in patients’ groups. These results could be explained by the assumption that the permanent inflammatory state of patients with silicosis facilitates, after contact with the antigen, the differentiation or maturation of virgin cells into memory T cells.

Regulatory T cells (Tregs) control and limit immune responses via the suppression of CD4^+^ and CD8^+^ T cells, playing a crucial role in the maintenance of immune homeostasis in the body under inflammatory conditions [36]. Our results revealed a notable increase in the Treg subset in silicosis patients compared with controls. Several studies on silicosis, both in vitro and in animal models, have described an increase in Treg cells [27,37,38], and targeted Treg depletion could be used as a therapeutic approach to attenuate the progression of this disease [39]. On the other hand, a decrease in Tregs has been observed in nonsilicosis CS-exposed workers preceding silicosis development [16]. This could be in line with our results considering that all our patients had been diagnosed with silicosis and had been developing the disease for years. In a rat animal model, after exposure to silica, the number of Treg cells initially decreased but increased over time and the disease progressed [37]. Additionally, a reduced number or function of Tregs has been described in a study with silicosis patients [40], but this discrepancy could be due to an age-corrected Treg percentage applied to the silicosis group in that study and to the imprecision of including as Treg cells a fraction with only two cell surface markers (CD4^+^CD25^+^) and at least a FoxP3^+^ or a CD127^−^ marker could be additionally used [20].

Currently, we know that natural killer (NK) cells not only have cytotoxic functions but also exert functions that influence innate and adaptive immunity and play immunoregulatory roles [41]. We tested whether the two main NK cell subsets in humans were altered in the peripheral blood of silicosis patients, but we did not observe any significant differences. The results reported in our study are comparable to those reported by other studies on other groups, including silica-exposed foundry and pottery workers (for whom data about their clinical status were unavailable) [13], hard rock miner workers with silicosis, and those with silica dust exposure without silicosis [42]. In both of these studies, total NK cells were analysed without a further analysis of NK cell subsets, resulting in the absence of any statistically significant difference in the number or percentage of NK cells in the peripheral blood. A more recent study [16] has described an increase in total NK cells in nonsilicosis CS-exposed workers, but this finding was not described in further detail. The discrepancy of this work with our data or with previous reports could be due to the selection of the NK cell fraction, which is not clearly defined in the methods section in that manuscript. However, a study reported no changes in NK cells in the bronchoalveolar lavage fluid, but not in peripheral blood, from silicosis patients [43]. Given the few results published on NK cell subsets in patients with silicosis, more studies are necessary to clarify the reported discrepancies.

In summary, patients with ES silicosis present a decrease in memory B lymphocytes, naïve T helper cells, and the CD4+/CD8+ ratio and an increase in regulatory T cells and plasmablasts. Compared to other chronic lung diseases with progressive fibrosis, a reduction in memory B cells was also observed in sarcoidosis [44,45,46] and systemic sclerosis (SSc) [47] but not in idiopathic pulmonary fibrosis (IPF) [48]. The plasmablast number has been reported not to be altered in SSc [47] or diminished in sarcoidosis [45,46] but is increased in IPF [49]. Regarding T cells, in sarcoidosis, as in our study, reduced naive CD4+ cells, increased CD8+ cells, and consequently a reduced CD4+/CD8+ ratio and an increase in Tregs were also observed [50,51,52,53]. No differences in CD4+ or CD8+ cells were observed in SSc and IPF. However, controversial results can be found in the subset of Tregs in IPF, where a reduction [54] or an increase [48] has been reported. In contrast to our results in patients with ES silicosis, where no differences in NK cell subsets were observed compared to healthy controls, an increase in total NK cells was observed in sarcoidosis, IPF, and SSc patients [47,55] although a decrease in IPF has been also reported [48].

## 4. Materials and Methods

### 4.1. Patient and Healthy Control Subjects

All patients included (*n* = 91) were male workers who were cutting, polishing, and finishing engineered stone countertops and were diagnosed with SS (*n* = 53) or with PMF (*n* = 38). They are part of a cohort of patients followed by the Pneumology Department of Puerta del Mar University Hospital in Cádiz (Spain). Patients had been diagnosed with silicosis based upon a history of exposure to silica and chest radiography and/or high-resolution computed tomography (HRCT) and, in some cases, by lung or mediastinal lymph node biopsy. Patients were asked to enrol in the study when they attended a hospital consultation. Respiratory function tests, chest radiographs, and HRCT scan classification of these patients have been described previously [15]. The exclusion criteria for patients in the study were active infection, kidney or liver disease, autoimmune rheumatic disease, or the use of immunosuppressive drugs; only oral corticosteroids use at a dose lower than 20 mg per day was accepted. Blood extraction was also performed on 22 healthy control (HC) subjects with no history of exposure to silica dust. All of them were hospital staff workers, and none of them had respiratory symptoms or chronic or acute disease. The medical evaluation before blood sampling was normal in all cases.

This study was approved by the institutional Research Ethics Committee of the province of Cadiz (registration No. 90.18, date 29 September 2018). All subjects provided written, informed consent following the Declaration of Helsinki. All the data were pseudonymized to preclude patient identification and were included in a database to which only the researchers had access.

### 4.2. Blood Sampling and Immune Profiling Panels

Venous blood samples were collected in 10 mL vacutainer tubes containing EDTA. Surface immunostaining of the cell population was performed by incubating 150 µL of whole peripheral blood for 15 min in the dark with the following antibodies in different combinations (Table S1): CD45-V500, CD3-APC-H7, CD4-V450, CD56-APC, CD19-PerCP-CY7, CD27-PE, CD16-FITC, CD45RA-FITC, and CD45RO-PE from BD (Becton Dickinson; San Jose, CA, USA); CD19-APC, CD38-FITC, and CD8-PerCP from Immunotools (Friesoythe, Germany); and CD127-PerCP-CY5.5 from BioLegend (San Diego, CA, USA). Subsequently, the peripheral blood was treated with 2 mL of lysing solution (ref. 349202, Becton Dickinson) for 5 min in the dark. The cells were washed and centrifuged before passage for cytometry. The different cellular populations (Table 2) were identified by using standard flow cytometry immunophenotyping techniques with a FACSCanto II cytometer (BD). At least 300,000 events were acquired for each sample.

### 4.3. Statistical Analysis

SPSS Statistics software, version 19.0 (IBM Corp., Armonk, NY, USA) was used for statistical analysis. Initially, the normality distribution of every set of data was established using the Kolmogorov–Smirnov test. Subsequently, one-way ANOVA for multiple (generally three: HC, SS, PMF) groups of data was performed by the ANOVA F test (normal distribution) or by the Kruskal–Wallis test (nonnormal distribution). For comparisons of two groups of data (HC vs. SS, HC vs. PMF, or SS vs. PMF), Student’s t-test (for normally distributed data) or the Mann–Whitney U test (for nonnormally distributed data) was used. The chi-square test was used to test relationships between categorical variables. The results are expressed as the mean and standard deviation (SD). A minimum significance level of *p* < 0.05 was adopted for all tests. Excluded data are from those that did not reach minimal detectable values to be included in a curve analysis and those considered extreme outliers (3.5 times above or below the mean value).

## 5. Conclusions

Taken together, our study revealed several peripheral blood immunological alterations that may play a role in the pathogenesis of silicosis. Chronic inflammatory and/or fibrotic processes in the lungs of ES-silicosis patients, even after prolonged cessation of exposure to silica, induce a decrease in memory B lymphocytes and naïve T helper cells and an increase in regulatory T cells and plasma cells. A better understanding of the role of these cell subsets will allow better control of immune system dysregulation and associated inflammation in these patients. Some limitations of our study are: (1) the cross-sectional nature of blood collection per patient, and, therefore, a longitudinal follow-up study of patients with silicosis to correlate changes in cell populations with the progression of the disease will be necessary; (2) it was not possible to quantify the environmental load of respirable silica dust over the years of exposure or accumulation of lung crystals in the study subjects; and (3) although all participants in the study were Caucasian and originally from the local surrounding areas, differences in genetic factors between individuals could influence the number of inflammatory cells.

## Figures and Tables

**Table 1:** Sociodemographic data of participants and pulmonary function values of patients with SS and PMF.

	HC (*n* = 22)	SS (*n* = 53)	PMF (*n* = 38)	*p*
Age *	36.4 ± 8.3	40.1 ± 7.7	41 ± 6.2	0.052 ^+^
Starting exposure age *	-	21.2 ± 7.4	21.4 ± 4.3	0.142 ^+^^+^
Duration of exposure *	-	13.1 ± 6.7	13.3 ± 6.1	0.968 ^+^^+^
Years since cessation of exposure to Blood draw *	-	6.4 ± 2.7	7.3 ± 2.5	0.058 ^+^^+^
Smoking status **				0.099 ^+^^+^^+^
Non-Smoker	15 (65.2)	22 (41.5)	15 (39.5)	
Ex-Smoker	4 (21.7)	26 (49.1)	21 (55.3)	
Smoker	3 (13)	5 (9.4)	2 (5.3)	
FEV_1_ (mL) *	nd	3386 ± 647	2961 ± 631	0.003
FEV_1_ (%) *	nd	87.8 ± 14	76.5 ± 14.8	<0.0001
FVC (mL) *	nd	4341 ± 748	3961 ± 783	0.022
FVC (%) *	nd	90.1 ± 13.3	82.3 ± 14.8	0.01
FEV_1_/FVC *	nd	0.77 ± 0.05	0.74 ± 0.07	0.009
DLCO (mmol/min/kPa) *	nd	9.2 ± 1.7	8.3 ± 1.4	0.006
DLCO (%) *	nd	85.4 ± 14.8	77.6 ± 14	0.014

Forced expiratory volume in 1 s (FEV_1_), forced vital capacity (FVC), diffusing capacity of lung for carbon monoxide (DLCO). * Mean ± standard deviation. ** Number of cases (percentages). ^+^ ANOVA F test, ^+^^+^ Mann–Whitney *U* test, ^+^^+^^+^ χ^2^ test; nd, not determined.

**Table 2:** Analysis of the surface markers of the lymphocyte subsets.

Surface Markers Tested	Immune Cell Phenotypes
CD19^+^	Total B cells
CD19^+^CD27^+^	Memory B cells
CD38^++^CD19^+/−^	Plasmablasts
CD3^+^	Total T cells
CD3^+^CD4^+^	Total helper T cells
CD3^+^CD8^+^	Total cytotoxic T cells
CD3^+^CD4^+^CD8^+^	Pro-T cells
CD3^+^CD4^−^CD8^−^	Naïve T cells
CD3^+^CD4^+^CD45RA^+^CD45RO^−^	Naïve helper T cells
CD3^+^CD4^+^CD45RA^−^CD45RO^+^	Memory helper T cells
CD3^+^CD4^+^CD25^++^CD127^−^	Regulatory T cells (CD4^+^)
CD3^−^CD56^+^	Total NK cells
CD3^−^CD56^dim^CD16^+^	Mature/Cytotoxic NK cells
CD3^−^CD56^bright^CD16^−^	Regulatory NK cells

## Data Availability

Existing ethical permits do not allow that personal data from this study are deposited in the public domain. The full dataset is available for researchers who meet the criteria for confidential data access as stipulated by participant informed consent and the Institutional Research Ethics Committee of the province of Cadiz (registration no. 90.18, date 29 September 2018), Spain.

## Supplementary Materials

The following supporting information can be downloaded at https://www.mdpi.com/article/10.3390/ijms25115722/s1.
