# Supplementary material for: Analysis of Immune Cell Subsets in Peripheral Blood from Patients with Engineered Stone Silica-Induced Lung Inflammation
Source: Int J Mol Sci. 2024 May 24;25(11):5722. doi: 10.3390/ijms25115722 (PMC11171478; doi:10.3390/ijms25115722)
Supplement: Supplementary file 1 [file ijms-25-05722-s001.zip › ijms-2964604-supplementary/Figure S1.pdf]

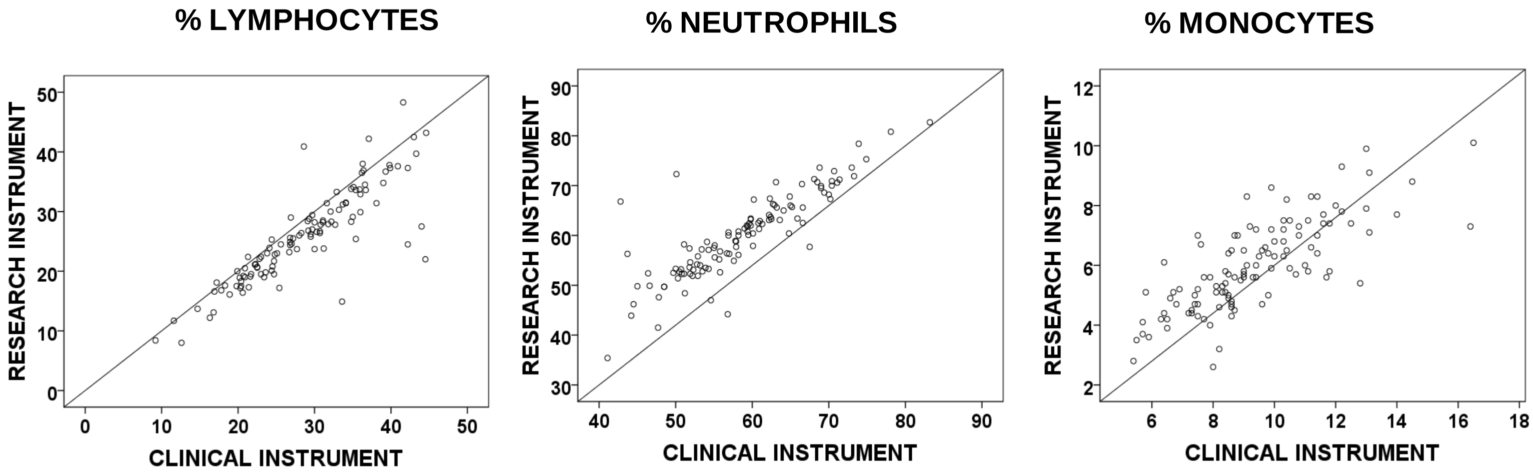

Figure S1. Correlation of the main leukocyte subsets measured by two instruments: a research instrument (Flow Cytometer) and a clinical instrument (Haematology Analyzer).
