# Supplementary material for: Analysis of Immune Cell Subsets in Peripheral Blood from Patients with Engineered Stone Silica-Induced Lung Inflammation
Source: Int J Mol Sci. 2024 May 24;25(11):5722. doi: 10.3390/ijms25115722 (PMC11171478; doi:10.3390/ijms25115722)
Supplement: Supplementary file 1 [file ijms-25-05722-s001.zip › ijms-2964604-supplementary/Table S1.pdf]

Table S1. Combinations and quantity of conjugated antibodies used for determinations of the cell subsets studied.

Combination 1			Combination 2			Combination 3		
Conjugated antibody	Quantity used per sample	Reference/ Manufacturer*	Conjugated antibody	Quantity used per sample	Reference/ Manufacturer*	Conjugated antibody	Quantity used per sample	Reference/ Manufacturer*
CD16-FITC	20 µl	335035 (BD)	CD45RA-FITC	20 µl	335039 (BD)	CD38 FITC	5 µl	21270383 (IT)
CD27-PE	20 µl	340425 (BD)	CD45RO-PE	20 µl	347967 (BD)	CD19-APC	5 µl	21270196 (IT)
CD8-PerCP	5 µl	21810085 (IT)	CD127-PerCP-Cy5.5	5 µl	351322 (BL)	CD45-V500	5 µl	560777 (BD)
CD19-PE-Cy7	5 µl	341113 (BD)	CD25-PE-Cy7	5 µl	335824 (BD)			
CD56-APC	5 µl	341027 (BD)	CD3-APC-H7	5 µl	560176 (BD)			
CD3-APC-H7	5 µl	560176 (BD)	CD4-V450	5 µl	651849 (BD)			
CD4-V450	5 µl	651849 (BD)	CD45-V500	5 µl	560777 (BD)			
CD45-V500	5 µl	560777 (BD)						

* (BD), Becton Dickinson; (IT), Immunotools; (BL), Biolegend
